# Characterization and Determination of Interesterification Markers (Triacylglycerol Regioisomers) in Confectionery Oils by Liquid Chromatography-Mass Spectrometry

**DOI:** 10.3390/foods7020023

**Published:** 2018-02-16

**Authors:** Valentina Santoro, Federica Dal Bello, Riccardo Aigotti, Daniela Gastaldi, Francesco Romaniello, Emanuele Forte, Martina Magni, Claudio Baiocchi, Claudio Medana

**Affiliations:** 1 Department of Molecular Biotechnology and Health Sciences, University of Turin, Turin 10124, Italy; federica.dalbello@unito.it (F.D.B.); riccardo.aigotti@unito.it (R.A.); daniela.gastaldi@unito.it (D.G.); francesco.romaniello@unito.it (F.R.); claudio.baiocchi@unito.it (C.B.); claudio.medana@unito.it (C.M.); 2 Soremartec Italy srl (Ferrero Group), AlbaCN 12051, Italy; Emanuele.FORTE@ferrero.com (E.F.); Martina.MAGNI@ferrero.com (M.M.)

**Keywords:** confectionery fats, positional isomers, silver-ion HPLC, triglycerides

## Abstract

Interesterification is an industrial transformation process aiming to change the physico-chemical properties of vegetable oils by redistributing fatty acid position within the original constituent of the triglycerides. In the confectionery industry, controlling formation degree of positional isomers is important in order to obtain fats with the desired properties. Silver ion HPLC (High Performance Liquid Chromatography) is the analytical technique usually adopted to separate triglycerides (TAGs) having different unsaturation degrees. However, separation of TAG positional isomers is a challenge when the number of double bonds is the same and the only difference is in their position within the triglyceride molecule. The TAG positional isomers involved in the present work have a structural specificity that require a separation method tailored to the needs of confectionery industry. The aim of this work was to obtain a chromatographic resolution that might allow reliable qualitative and quantitative evaluation of TAG positional isomers within reasonably rapid retention times and robust in respect of repeatability and reproducibility. The resulting analytical procedure was applied both to confectionery raw materials and final products.

## 1. Introduction

There is a great demand from food industry of fats with different degrees of solidity. However, most of the fats coming from natural sources are oils so they need to be suitably transformed in order to modulate their hardness. Interesterification, by changing the position of fatty acids within the original triglyceride structure, is the process more commonly used to modify consistency of oils [1]. Such result may be obtained either chemically using a catalyst or by the use of enzymes. In both cases, positional isomers (regioisomers) from original triglycerides are formed and the amount of their formation modulates the final physical properties of the fat.

TAGs (triglycerides) composition of a fat is perceived by the consumer since it is associated with the spreading properties, hardness and palatability of the product.

Small variations in TAG structure may have an impact on crystallization and polymorphic properties of fats. Thermal processes applied for technological reasons (e.g., deodorization) may produce positional isomers, inducing changes in the physical characteristics of fats. In fact, deodorization at high temperatures induces formation of asymmetric TAGs influencing crystallization behavior. The above changes must be monitored by analytical methods and kept under control in the course of the industrial process in relation to quality control and research and development of new TAGs composition that can improve product quality.

Among natural fats, cocoa butter has a particular relevance because of its unusual and highly valued physical properties and its peculiar triglycerides’ content. They are symmetrical monounsaturated TAGs containing three major fatty acids: palmitic (P), stearic (S) and oleic (O) and three main glycerides, palmitoyl-oleoyl-palmitoyl glycerol (POP), palmitoyl-oleoyl-stearoyl glycerol (POS) and stearoyl-oleoyl-stearoyl glycerol (SOS), with the unsaturated fatty acid located in the central position as it is typical in vegetable oils.

If thermal process for deodorization is applied to cocoa butter, positional isomers may be produced with a change in the physical characteristics of the product. As already said, deodorization at high temperatures induces a significant formation of asymmetric TAGs influencing the crystallization behavior.

The main analytical technique for regiospecific analysis of TAGs is silver-ion high performance liquid chromatography (HPLC) coupled via an atmospheric pressure chemical ionization (APCI) source to a mass (MS) detector. Silver ion chromatography is based on the interaction of silver ions, bounded to a cation-exchange stationary phase, with π electrons of double bonds of unsaturated TAGs [2,3]. However, even if silver-ion HPLC remains the unique separation technique of unsaturated fats, its intrinsically difficult use in terms of repeatability and reproducibility must be underlined. Generally, the equilibration times are long and separations in gradient conditions are not easily reproducible.

In addition, conditioning times between successive analyses are time-consuming due to the necessary transition between two mobile phase compositions having different strength.

Furthermore, separation in isocratic conditions does require daily, before starting a sequence, long conditioning times and blank chromatographic runs to attain reproducibility. Separation of TAGs containing fatty acids having different unsaturation degrees by silver-ion HPLC has been the object of several publications [4,5,6,7,8,9,10,11,12,13,14,15,16,17,18].The aim of many of them was to obtain a detailed qualitative profile of complex mixtures of animal and vegetable fats. However, baseline separation of variously unsaturated TAGs, necessary for reliable quantitative analyses, was not always achieved because of the matrix complexity. In many cases, when three columns in series were used, retention times were inevitably long [15,16].

More recently, the use of traditional reversed phase column (polymeric bonded phase) was reported. However, the separation of critical regioisomer couples not always was enough to assure accurate quantitative evaluation of each component and their retention times were also long [19].

An interesting recent work [20] presents a rapid separation of TAG regioisomers in animal fats by Differential Mobility Spectrometry (DMS). However, the optimization of various experimental parameters such as the separation and compensation voltages applied to DMS electrodes, the type and flow rate of chemical modifier and the dwell time of analyte ions in the DMS cell is required. In addition, to be expensive, the technique is sophisticated and scarcely suitable to the analytical skill generally present in industry internal quality control laboratory.

The aim of the present paper is to describe a separation method of individuated typical TAGs of cocoa butter, palm and other tropical oils mainly used in confectionery industry. The main couples of triacylglycerol regioisomers that had to be separated, identified and quantified were, respectively, POP/PPO (palmitoyl-palmitoyl-oleoyl glycerol), POS/PSO (palmitoyl-stearoyl-oleoyl glycerol, SOS/SSO (stearoyl-stearoyl-oleoyl glycerol) and POO/OPO (palmitoyl-oleoyl-oleoyl glycerol/oleoyl- palmitoyl-oleoyl glycerol), SOO/OSO (stearoyl-oleoyl-oleoyl glycerol/oleoyl-stearoyl-oleoyl glycerol).

The chromatographic problem we had to face up to was challenging because we needed to obtain retention times as low as possible and selective enough to allow a reliable peak integration. A task not easy to obtain because we had two classes of compounds with very different retention times due to a different number of double bonds (e.g., POP in respect to POO, etc.) and with a very subtle structural difference within the single class (different position of an oleic acid residue within the triglyceride structure). Separation of the regioisomer couples before cited, reported in literature, was characterized by very long retention times (40–60 min) [14,15,16] not compatible with the analysis times useful for a confectionery industry internal control laboratory.

As a matter of fact, the high chromatographic selectivity necessary to separate the regioisomers belonging to the same class is conflicting with the quite lower selectivity required to separate the constituents of the two classes. In other words, higher selectivity conditions (elution phase of low force) necessary to separate the couples POP/PPO, POS/PSO and SOS/SSO would cause the species POO/OPO and SOO/OSO to have very different and longer retention times. As a consequence, we will try to find a compromise and, differently from the works reported in literature, we used cation exchange columns silver modified by us in order to achieve a better control on retention mechanism by varying, if necessary, the degree of silver modification of the stationary phase.

Due to the small number of TAG isomers to be separated belonging to two classes of molecules differing by the number of double bonds, we adopted two different isocratic steps and a rapid transition between them as a more controllable separation mode.

## 2. Materials and Methods 

### 2.1. Reagents

Isopropanol, acetonitrile, *n*-heptane and ethyl acetate LC-MS (Liquid Chromatography-Mass Spectrometry)-grade solvents, POP, PPO, POS, PSO, SOS, SSO, OPO, POO, SOO, and OSO TAG pure standards and anhydrous Na_2_SO_4_ were purchased from Sigma-Aldrich (St. Louis, MO, USA).

### 2.2. Sample Preparation

Samples obtained from confectionery industry were previously heated to complete fusion and homogenized by mechanical stirring. Successively were dehydrated with Na_2_SO_4_ and filtered through Buchner. A sample aliquot (10.0 mg) was added to of *n*-heptane (10 mL) and analyzed after suitable dilution.

### 2.3. Calibration Curve Preparation

A stock solution of all standards mixed together was prepared in *n*-heptane at a concentration of 1000 ppm each. To draw a calibration curve, the stock solution was diluted to contain 1.0, 2.0, 3.0, 5.0, 8.0, 10.0 ppm, respectively.

### 2.4. Instrumentation

The separation system was a binary solvent HPLC Ultimate 3000 (Thermo Fisher Scientific, Milan, Italy) interfaced through an APCI ionization source to a linear ion trap coupled to a high resolution mass analyzer (LTQ-Orbitrap Thermo Fisher Scientific, Milan, Italy). APCI ionization source heated at 450 °C was used in positive ions mode in the mass range 240–1000 Th.

Capillary temperature was 250 °C, flow rate of sheath gas and auxiliary gas were set at 35.0 and 15.0 arbitrary units, capillary voltage was 25.0 V, source voltage 6.0 kV, and tube lens 110.0 V. Mass resolution was set at 30,000.

### 2.5. Chromatography

A cation exchange column (Luna SCX, Phenomenex, 150 × 2.0 mm, 5 µm, 100 Å) silver-modified by us was used. A gradient separation made up by two isocratic steps at different mobile phase compositions separated by a rapid transition time of five minutes between them was used. The two mobile phase compositions (*n*-heptane:ethylacetate 93:7 and 90:10, respectively) were chosen according to elution times and peak resolution. Flow rate was 0.300 mL/min. The injection volume was 10.0 µL.

### 2.6. Silver Modification of SCX Columns

After the inversion of the flow direction, we rinsed the column with methanol/acetonitrile (CH_3_OH/CH_3_CN) 1:1 for about 45–60 min (flow rate 1.0 mL/min). As the concentration of AgNO_3_ solution and the number and volume of injections determine the retention characteristic of the modified SCX stationary phase, we tested three concentrations of AgNO_3_ solution (0.57 M, 1.18 M and 1.86 M). During the rinsing procedure, ten 50.0 µL injections of a 1.18 M AgNO_3_ solution in CH_3_CN allowed for obtaining a chromatographic result suited to our requirements. The column was successively inverted to the original position and rinsed with dichloromethane for an hour at a flow rate of 0.20 mL/min.

### 2.7. Statistical Evaluation of Final Data and Validation

The analytical procedure, the object of the present work, is destined to be applied to raw material and to final confectionery products (interesterified or not), so real samples of whatever concentration may be at one’s disposal. However, validation parameters like Limit of Detection (LOD) or Limit of Quantitation (LOQ) were anyway evaluated. Very important for our purposes are parameters like linearity, measure uncertainty, repeatability and reproducibility. Their definition is assuming particular significance in the present analytical context because taking under control the mobile and stationary phase conditions ruling the chromatographic performances was a particularly demanding task as described before.

In addition, as already stated, a determination of the amount of positional isomers, as accurate as possible, is functional to establish a reliable relationship with the interesterification conditions.

A calibration curve was realized in six replicates and each curve was made by six points spanning from 1.0 to 10.0 ppm. The straight line was calculated by a linear regression method and each calibration point was the mean of the six replicates. The *R*^2^ value was used as a first linearity evaluator. The successive statistical calculations were based on the evaluation of the mean calibration curve error. Hubaux-Vos formulae were applied [21].

In Table 1, all data concerning linearity, measure uncertainty, repeatability, reproducibility, LOD and LOQ for the positional isomers object of this study are reported along with the mean calibration curves for the standards.

## 3. Results and Discussion

In literature, there are no papers devoted explicitly to specific regioisomers separation. They are concerning characterization of triacylglycerol composition in animal fats [13] or complex natural triacylglycerol mixture [15]. General works regarding the effect of temperature and mobile phase composition [12] on regioisomer separation involved triacylglycerol positional isomers very different from those envisaged in the present work. Anyway, when couples like POP/PPO or SOS/SSO, important in our case, were occasionally present in some of the reported separations in literature, they showed retention times very long (between 40 and 60 min) and the obtained chromatographic selectivity was due to the use of three columns in series. Such separation conditions are incompatible with the typical exigencies of industrial analytical laboratories. Thus, we had to reformulate the separation in a manner more tailored to the molecular characteristics of the specific TAG regioisomers of interest in confectionery industry and to the performance of their internal laboratories.

As a matter of fact, the choice of chromatographic separation conditions characterized by two isocratic steps reported in the Materials and Methods section was the result of a fine-tuning of the mobile phase strength. The mobile phase composition must be strong enough to assure reasonably short retention times and, at the same time, not too strong to level out the chromatographic resolution based on different stereochemistry of TAG positional isomers. 

At first, we checked the difference in selectivity between two elution strength modifiers like acetonitrile and ethyl acetate, which have a very different elution force. Mixtures of *n*-hexane or *n*-heptane and a very small percent of acetonitrile (0.8–0.11%) provide good separations of unsaturated TAGs [3,4,5]. In these cases, acetonitrile concentration must be very low due to its high elution force and its poor miscibility with *n*-hexane or *n*-heptane. Such a mixture is unstable and tends to give chromatographic results not easily reproducible. The HPLC binary pump was unsatisfactory in the delivery of the mixed mobile phase because of its low mixing equilibrium; consequently, pre-mixing was necessary. Sometimes, in order to overcome this problem, isopropanol is used to intermediate the two immiscible solvents [11,12,13,14,15,16,17,18]. The equilibrium of the mixture was always precarious. Subtle differences in mixture preparation, temperature oscillations and different rates of unavoidable evaporation from the HPLC reservoirs of the three mobile phase components were all factors that often had a substantial influence on retention times. Maintaining time elution reproducibility was a challenge, and day-by-day chromatographic variations were recorded, making conditioning times progressively longer. All of these troubles are perfectly managed in academic studies where careful attention to difficult experimental conditions can be paid. On the contrary, in the case of internal laboratories of confectionery industry, a more easily applicable protocol would be at one’s disposal.

Alternatively, the use of ethyl acetate as a modifier of *n*-heptane allowed for obtaining slightly better reproducible chromatography, conditioning times shorter and by far better results’ reproducibility. 

Different elution conditions were also tested and eventually a satisfactory chromatographic resolution was obtained with two successive isocratic segments involving *n*-heptane:ethyl acetate compositions of 93:7 and 90:10, respectively, with five minutes to change from the initial to final composition. In addition, only one column was used to obtain a satisfactory separation.

**Figure 1:**
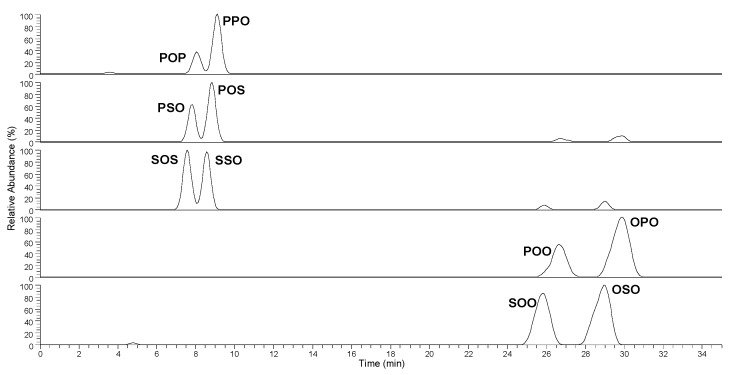
Chromatographic separation of standard TAG (triglyceride) regioisomers visualized at the extracted precursor ion m/z values.

In Figure 1, the separation of standard samples of regioisomeric couples POP/PPO, POS/PSO, SOS/SSO, POO/OPO and SOO/OSO, at the same concentration (10.0 ppm), monitored by a linear ion trap is shown. 

As it can be seen, the chain length of TAG fatty acids has extremely limited influence on the retention times. The different spatial position of the double bond of oleic acid in sn-2 with respect to sn-1/sn-3 positions is enough to produce separation of regioisomers with the same number of double bonds whereas the presence of two double bonds change drastically the retention times. 

The MS analyser provided structural information already coming from the thermal lability of triglycerides in the hot (450 °C) APCI ionization source. Triglycerides tend to release their constituent fatty acids in a typical way involving primarily the ones in the external position and to a lesser extent the one in the central position [22]. This behavior allows for distinguishing the isobaric couples of positional isomers.

**Figure 2:**
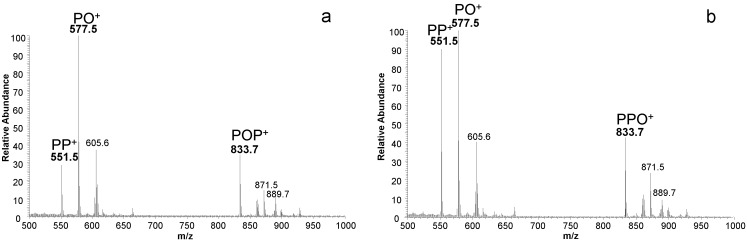
Full mass spectra of isobaric positional isomers POP (palmitoyl-oleoyl-palmitoyl glycerol) (**a**) and PPO (palmitoyl-palmitoyl-oleoyl glycerol) (**b**) showing the specificity of thermal fragmentation mechanism.

An example of full mass spectra of standard TAG positional isomers POP/PPO is shown in Figure 2a,b. The qualitative pattern of thermal product ions is very similar but with different signal intensity as a consequence of the selective fragmentation pathway privileging the loss of fatty acids in external positions.

**Figure 3:**
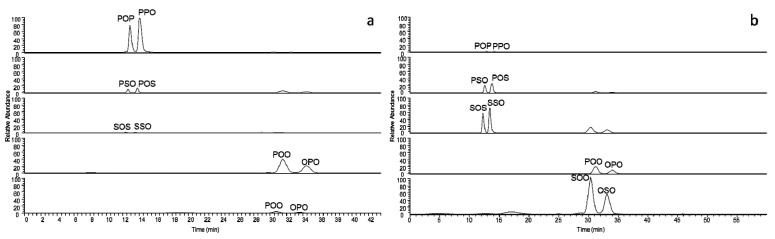
Chromatographic separation of TAG regioisomers typical of interesterified Shea oil (**a**) and Palm oil (**b**) visualized at the extracted precursor ion m/z values.

By applying the same experimental conditions to interesterified samples obtained from the confectionery industry, shea oil or palm olein, it is possible to obtain evidence of their interesterification degree, as shown in the chromatographic profiles of Figure 3a,b. As expected, the signal intensity of the different couples of regioisomers in the two oils reflects their TAG composition, in particular the low presence of palmitic acid in shea oil TAG constituents.

**Figure 4:**
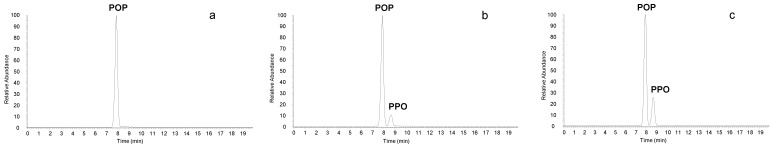
LC-MS (Liquid chromatography-mass spectrometry) chromatograms of cocoa butter samples pre deodorization (**a**) and deodorized at two different temperatures 220 °C (**b**) and 260 °C (**c**), corresponding to the absence and to the presence of thermally formed positional isomers, respectively.

Figure 4a–c shows LC-MS chromatograms of a cocoa butter samples pre and after deodorization at two different temperatures (220 °C and 260 °C) corresponding to the increasing presence of thermally formed positional isomers, respectively (as an example, only the couple POP, PPO is reported).

In addition to confectionery industry raw materials, samples of final products were analyzed. In Table 2, Table 3 and Table 4, meaningful results are reported about a commercial biscuit filling cream and two spreading creams. As it can be seen, there is evidence of TAG regioisomer formation in two final products with respect to the original raw materials used.

Thus, it was possible to establish a reliable relationship between the extent of positional isomers formation and the experimental conditions to which industry samples can undergo. In this way, on the one hand, undesirable regioisomer formation may be avoided and, on the other, a regioisomer formation may be guided until the achievement of the desired physico-chemical properties. 

## 4. Conclusions

Interesterification process of vegetable oils is of paramount importance in the confectionery industry because positional isomer formation allows for modulating the fat property. On the other hand, unwanted interesterification events may occur during thermal treatment of fats as in the deodorizing process. Therefore, either in the case of intentionally producing an interesterification process or checking its unwanted occurrence, it is necessary to have at one’s disposal an analytical method helping to establish a sound relationship between the extent of positional isomer presence and the experimental conditions causing their formation. 

In this work, we developed an analytical protocol based on LC-MS analysis able to identify and quantify the positional isomers typical of confectionery fats undergoing the interesterification process or thermal treatment. The time required for the global analysis was relatively short, the chromatographic resolution and efficiency were satisfactory and the mass detection allowed for identifying the isobaric components of each positional isomer couple. 

In conclusion, the method described may well be considered a good diagnostic tool of interesterification consequences that are strictly connected to confectionery product quality.

## Figures and Tables

**Table 1:** Validation data: calibration curve parameters, repeatability, reproducibility, uncertainty measure, limit of detection (LOQ) and limit of quantification (LOQ).

Tag	Calibration Points (ppm)	Calibration Curve Parameters ^1^	Rsd (%)	Calibration Curve Parameters ^2^	Rsd (%)	Measure Uncertainty x_0_ ± *t* (0.05, 5)s_x0_	LOD, LOQ (ppm)
SOS	1	*b* = (5.91 ± 0.06) × 10^6^ *R*² = 0.9996	13.9	*b* = (5.82 ± 0.10) × 10^6^ *R*² = 0.9985	10.3	1.00 ± 0.38	0.31, 1.04
	2		6.73		8.85	1.89 ± 0.34	
	3		5.99		6.81	2.87 ± 0.32	
	5		6.39		5.09	5.01 ± 0.29	
	8		2.13		7.90	7.61 ± 0.34	
	10		1.22		2.25	10.37 ± 0.46	
SSO	1	*b* = (7.79 ± 0.07) × 10^6^ *R*² = 0.9996	2.42	*b* = (7.84 ± 0.10) × 10^6^ *R*² = 0.9992	10.6	0.97 ± 0.27	0.31, 1.03
	2		15.5		9.96	2.19 ± 0.24	
	3		6.46		5.65	2.91 ± 0.22	
	5		4.01		5.71	4.69 ± 0.21	
	8		1.82		2.30	8.09 ± 0.25	
	10		1.09		9.86	10.1 ± 0.31	
POS	1	*b* = (5.76 ± 0.07) × 10^6^ *R*² = 0.9995	13.9	*b* = (5.61 ± 0.04) × 10^6^ *R*² = 0.9997	9.56	1.06 ± 0.16	0.30, 1.01
	2		6.03		5.10	2.17 ± 0.14	
	3		2.12		4.28	3.13 ± 0.13	
	5		4.13		5.18	4.91 ± 0.12	
	8		1.69		4.28	7.97 ± 0.15	
	10		8.64		10.6	9.99 ± 0.18	
PSO	1	*b* = (9.34 ± 0.10) × 10^6^ *R*² = 0.9985	5.22	*b* = (9.41 ± 0.09) × 10^6^ *R*² = 0.9995	9.86	1.02 ± 0.21	0.30, 1.00
	2		7.16		8.57	2.03 ± 0.19	
	3		3.55		4.41	2.82 ± 0.17	
	5		0.45		3.52	4.96 ± 0.16	
	8		3.64		3.78	7.85 ± 0.19	
	10		4.05		6.86	10.2 ± 0.25	
POP	1	*b* = (3.69 ± 0.06) × 10^6^ *R*² = 0.9989	7.76	*b* = (3.61 ± 0.05) × 10^6^ *R*² = 0.9992	9.02	0.95 ± 0.27	0.26, 0.85
	2		4.89		7.48	2.14 ± 0.24	
	3		5.11		11.1	3.16 ± 0.22	
	5		9.37		11.3	4.84 ± 0.21	
	8		4.73		5.28	7.99 ± 0.25	
	10		2.81		12.5	10.1 ± 0.32	
PPO	1	*b* = (12.60 ± 0.07) × 10^6^ *R*² = 0.9998	11.1	*b* = (12.67 ± 0.06) × 10^6^ *R*² = 0.9999	10.02	1.07 ± 0.11	0.32, 1.07
	2		1.97		4.38	2.02 ± 0.10	
	3		4.59		4.97	3.04 ± 0.09	
	5		0.75		4.59	4.87 ± 0.08	
	8		3.42		5.14	7.99 ± 0.10	
	10		5.38		9.52	10.1 ± 0.12	
SOO	1	*b* = (16.19 ± 0.21) × 10^6^ *R*^2^ = 0.9992	6.10	*b* = (16.26 ± 0.27) × 10^6^ *R*² = 0.9960	9.10	1.19 ± 0.35	0.32, 1.08
	2		4.13		2.62	2.07 ± 0.32	
	3		2.29		2.13	2.91 ± 0.30	
	5		0.71		8.05	5.38 ± 0.28	
	8		1.73		1.80	8.10 ± 0.34	
	10		1.96		4.74	9.72 ± 0.40	
OSO	1	*b* = (22.37 ± 0.25) × 10^6^ *R*^2^ = 0.9994	0.99	*b* = (22.31 ± 0.23) × 10^6^ *R*² = 0.9990	4.25	1.20 ± 0.22	0.15, 0.51
	2		2.94		1.95	2.14 ± 0.20	
	3		1.56		2.65	3.11 ± 0.18	
	5		3.89		5.58	4.90 ± 0.17	
	8		3.49		4.66	7.86 ± 0.20	
	10		0.45		3.79	10.1 ± 0.26	

^1^ Repeatibility; mean of three replicates; ^2^ reproducibility; mean of six replicates.

**Table 2:** Comparison of TAG (triglyceride) percent composition between original raw materials and confectionery final products (biscuit filling cream).

TAG	Biscuit Filling Cream TAG Composition (%)	Original Raw Material	
		Shea Butter (%)	High Oleic Rapeseed Oil (%)
POP	84.1	91.51	100
PPO	15.9	8.49	0
POS	77.3	100	100
PSO	22.7	0	0
SOS	80.3	100	100
SSO	19.7	0	0
POO	100	100	100
OPO	0	0	0
SOO	88.2	100	100
OSO	11.8	0	0
Evidence of interesterification			

**Table 3:** Comparison of TAG percent composition between original raw materials and confectionery final products (spreading cream A).

Tag	Spreading Cream A TAG Composition (%)	Original Raw Material	
		High Linoleic Sunflower Oil (%)	Cocoa Butter (%)
POP	95.7	100	100
PPO	4.3 ^1^	0	0
POS	100	100	100
PSO	0	0	0
SOS	100	100	100
SSO	0	0	0
POO	100	100	100
OPO	0	0	0
SOO	100	100	100
OSO	0	0	0
No interesterification			

^1^ Coming from vaccine butter fraction present in milk powder.

**Table 4:** Comparison of TAG percent composition between original raw materials and confectionery final products (spreading cream B).

TAG	Spreading Cream BTAG Composition (%)	Original Raw Material	
		High Linoleic Sunflower Oil (%)	Cocoa Butter (%)
POP	83.0	100	100
PPO	17.0	0	0
POS	84.5	100	100
PSO	15.5	0	0
SOS	84.1	100	100
SSO	15.9	0	0
POO	91.7	100	100
OPO	8.3	0	0
SOO	91.7	100	100
OSO	8.3	0	0
Evidence of interesterification			

## References

[1] Gupta M.K. (2010). Practical Guide to Vegetable Oil Processing.

[2] De Vries B. (1962). Quantitative separation of lipid materials by column chromatography on silica impregnated with silver nitrate. Chem. Ind. J..

[3] Adlof R.O., Menzel A., Dorovska-Taran V. (2002). Analysis of conjugated linoleic acid-enriched triacylglycerol mixtures by isocratic silver-ion high-performance liquid chromatography. J. Chromatogr. A.

[4] Christie W.W. (1988). Separation of molecular species of triacylglycerols by high-performance liquid chromatography with a silver ion column. J. Chromatogr. A.

[5] Adlof R.O. (1995). Analysis of triacylglycerol positional isomers by silver ion high-performance liquid chromatography. J. High Resolut. Chromatogr..

[6] Adlof R.O. (1997). Normal-phase separation effects with lipids on a silver ion high-performance liquid chromatography column. J. Chromatogr. A.

[7] Laakso P., Voutilainen P. (1996). Analysis of triacylglycerols by silver-ion high-performance liquid chromatography—Atmospheric pressure chemical ionization mass spectrometry. Lipids.

[8] Schuyl P.J.W., de Joode T., Vasconcellos M.A., Duchateau G.S.M.J.E. (1998). Silver-phase high-performance liquid chromatography–electrospray mass spectrometry of triacylglycerols. J. Chromatogr. A.

[9] Mondello L., Tranchida P.Q., Stanek V., Jandera P., Dugo G., Dugo P. (2005). Silver-ion reversed-phase comprehensive two-dimensional liquid chromatography combined with mass spectrometric detection in lipidic food analysis. J. Chromatogr. A.

[10] Dugo P., Kumm T., Crupi M.L., Cotroneo A., Mondello L. (2006). Comprehensive two-dimensional liquid chromatography combined with mass spectrometric detection in the analyses of triacylglycerols in natural lipidic matrixes. J. Chromatogr. A.

[11] Nikolova-Damyanova B. (2009). Retention of lipids in silver ion high-performance liquid chromatography: Facts and assumptions. J. Chromatogr. A.

[12] Lisa M., Velínská H., Holčapek M. (2009). Regioisomeric Characterization of Triacylglycerols Using Silver-Ion HPLC/MS and Randomization Synthesis of Standards. Anal. Chem..

[13] Lisa M., Netusilova K., Franek L., Dvorakova H., Vrkoslav V., Holcapek M. (2011). Characterization of fatty acid and triacylglycerol composition in animal fats using silver-ion and non-aqueous reversed-phase high-performance liquid chromatography/mass spectrometry and gas chromatography/flame ionization detection. J. Chromatogr. A.

[14] Lisa M., Denev R., Holčapek M. (2013). Retention behavior of isomeric triacylglycerols in silver-ion HPLC: Effects of mobile phase composition and temperature. J. Sep. Sci..

[15] Holčapek M., Velínská H., Lísa M., Česla P. (2009). Orthogonality of silver-ion and non-aqueous reversed-phase HPLC/MS in the analysis of complex natural mixtures of triacylglycerols. J. Sep. Sci..

[16] Holčapek M., Dvorakova H., Lisa M., Giron A.J., Sandra P., Cvacka J. (2010). Regioisomeric analysis of triacylglycerols using silver-ion liquid chromatography atmospheric pressure chemical ionization mass spectrometry: Comparison of five different mass analyzers. J. Chromatogr. A.

[17] Byrdwell W.C., Holčapek M. (2011). Extreme Chromatography: Faster, Hotter, Smaller.

[18] Ovčačíková M., Lisa M., Cifkova E., Holcapek M. (2016). Retention behavior of lipids in reversed-phase ultrahigh-performance liquid chromatography-electrospray ionization mass spectrometry. J. Chromatogr. A.

[19] TambaSompila A.W.G., Héron S., Hmida D., Tchapla A. (2017). Fast non-aqueous reversed-phase liquid chromatography separation of triacylglycerol regioisomers with isocratic mobile phase. Application to different oils and fats. J. Chromatogr. B.

[20] Šala M., Lísa M., Campbell J.L., Holčapek M. (2016). Determination of triacylglycerol regioisomers using differential mobility spectrometry. Rapid Commun. Mass Spectrom..

[21] Hubaux A., Vos G. (1970). Decision and detection limits for linear calibration curves. Anal. Chem..

[22] Baiocchi C., Medana C., Dal Bello F., Giancotti V., Aigotti R., Gastaldi D. (2015). Analysis of regioisomers of polyunsaturated triacylglycerols in marine matrices by HPLC/HRMS. Food Chem..

